# Digitalization of education and mental health

**DOI:** 10.1192/j.eurpsy.2021.937

**Published:** 2021-08-13

**Authors:** R. Shilko, S. Egorov, Y. Zinchenko

**Affiliations:** Faculty Of Psychology, Lomonosov Moscow State University, Moscow, Russian Federation

**Keywords:** mental health, Education, digitalization

## Abstract

**Introduction:**

Various social problems, financial difficulties and academic factors contribute to the fact that more and more students around the world experience mental health problems (Chen et al., 2013; Gotlib et al., 2019). On the other hand, all the sides of students’ lives – from education to family relationships – are mediated by information communication technologies, that may have broad and ambiguous influence on students mental health. What is undoubtedly that youth mental health can no longer be considered without touching on the digitalization, including in education.

**Objectives:**

The current study aims to point up positive and negative examples of intersection of education digitalization and mental health of modern youth.

**Methods:**

Theoretical analysis of research publications and conceptualization of practical applications in education mediated by digital technologies.

**Results:**

A striking example of the negative impact of total digitalization of education was the sharp deterioration in mental health in the context of the transition to fully distant learning in conditions of the spread of coronavirus infection. Positive education as teaching form for both traditional skills and positive functioning and happiness provides a broad opportunities to combine the concepts of positive psychology with cutting-edge high-tech education approaches.

**Conclusions:**

Digitalization of modern education can be accompanied by both mental health risks and new opportunities. Mental health support can be based on finding information about good functioning, learning and participating in community activities that are provided through websites and mobile applications. The reported study was funded by the Russian Foundation for Basic Research, project number 18-29-22049.

